# A framework for normalized extraction of fine-grained traditional Chinese medicine symptom entities and relations

**DOI:** 10.1186/s12911-025-03257-4

**Published:** 2025-12-06

**Authors:** Xingyue Gou, Junyu Yao, Wei Lai, Yuzhu Gao, Siqi Wang, Chuangan Zhou, Hui Ye, Jing Tian, Jun Yi, Dong Cao

**Affiliations:** 1 School of Medical Information Engineering, Guangzhou University of Chinese Medicine, Guangzhou, Guangdong Province 510006 China; 2 Yukang School of Medicine and Health, Guangzhou Nanfang College, Guangzhou, Guangdong Province 510970 China; 3 School of Foreign Studies, Guangzhou University of Chinese Medicine, Guangzhou, Guangdong Province 510006 China; 4 School of Medical Information Engineering, Guangdong Pharmaceutical University, Guangzhou, Guangdong Province 510006 China

**Keywords:** Symptom information extraction, TCM EMRs, Symptom normalized output, Fine-grained annotation guideline, Normalized corpus construction

## Abstract

**Background:**

In Traditional Chinese Medicine Electronic Medical Records (TCM EMRs), symptom descriptions are often semi-structured, and coarse-grained annotation can lead to symptom nesting and information loss. To address these limitations and improve the precision of symptom representation, this study proposes a fine-grained symptom entity annotation system. Its objective is to convert unstandardized symptom expressions into structured data, thereby enhancing the correlation and standardization of symptom information to support intelligent TCM diagnosis and treatment.

**Methods:**

A five-step approach was employed: First, we drafted a fine-grained annotation guideline based on existing research. Second, we annotated symptom entities and iteratively refined the annotations through trial runs, multiple revisions, and evaluations. Third, we trained and assessed Named Entity Recognition (NER) models. Fourth, we extracted relations using predefined rules. Finally, we generated normalized outputs by integrating these rules and manually validated the extraction results.

**Results:**

The study annotated 500 TCM EMRs over five trials, identified 12 entity categories and 10 relation types. The inter-annotator agreement (IAA) F1 scores for entities and relations were 93.56% and 91.23%, respectively. The final corpus comprises 39,097 entities and 41,373 relation pairs, with 15,853 normalized symptom sentences generated through rule-based combination. Compared to prior studies, TCM symptom information utilization (TCM-SIU) increased by 8.24%. The best-performing NER model achieved an F1 score of 92.29%, while rule-based Relation Extraction (RE) attained an F1 score of 88.17%.

**Conclusion:**

The proposed fine-grained symptom annotation system significantly enhances the utilization of symptom information. It effectively mitigates symptom nesting issues, supports comprehensive association, and facilitates structured output, thereby providing robust data for symptom standardization and precise syndrome differentiation.

**Supplementary Information:**

The online version contains supplementary material available at 10.1186/s12911-025-03257-4.

## Background

One of the treasures of Traditional Chinese Medicine (TCM) is its diagnostic system, which relies on thorough symptom observation and analysis. For an accurate diagnosis and treatment, leveraging symptom information is crucial [1, 2]. With the rapid advancement of artificial intelligence (AI), intelligent-assisted diagnosis and treatment in TCM have become a significant trend in the medical field [1, 3]. As a key component of TCM smart healthcare development, the volume of TCM Electronic Medical Records (TCM EMRs) has surged, providing a wealth of data for TCM corpus collection and analysis. Consequently, a primary focus of current TCM EMR data mining research is extracting valuable symptom information [4].

In recent years, research on data mining of electronic medical records (EMRs) makes substantial progress, with various approaches being developed for medical information extraction. In TCM EMRs, symptom information is especially abundant and essential. However, the absence of a unified national symptom standard leads to diverse and complex descriptions, resulting in a large proportion of unstructured data [5]. This complexity increases the difficulty of symptom extraction and reduces the accuracy of symptom recognition.

Although preliminary efforts are made to extract symptom information, most existing studies treat symptoms, diseases, and medications as entities of the lowest dimension for extraction, which may lack other clinically relevant dimensional information—such as symptom onset time, contextual conditions, and severity [6, 7]. However, according to the principles of TCM syndrome differentiation and treatment determination, these factors significantly influence clinical interpretation. Ignoring these fine-grained elements undermines the precision of symptom analysis and fails to support accurate TCM diagnosis [8, 9]. Zhang et al. [5] construct a fine-grained TCM corpus focused on body part entities, achieving high annotation consistency (IAA = 0.936) for entity recognition. Pokharela et al. [10] develop a fine-grained Temporal Tree representation combined with doc2vec to improve patient similarity computation in Intensive Care Unit (ICU) Electronic Health Records. In other areas of medicine, Ma et al. [11] further validate the utility of fine-grained features through a multi-modal pre-training model (M2CG), enhancing skin disease diagnosis accuracy by up to 11.11%.

**Fig. 1:**
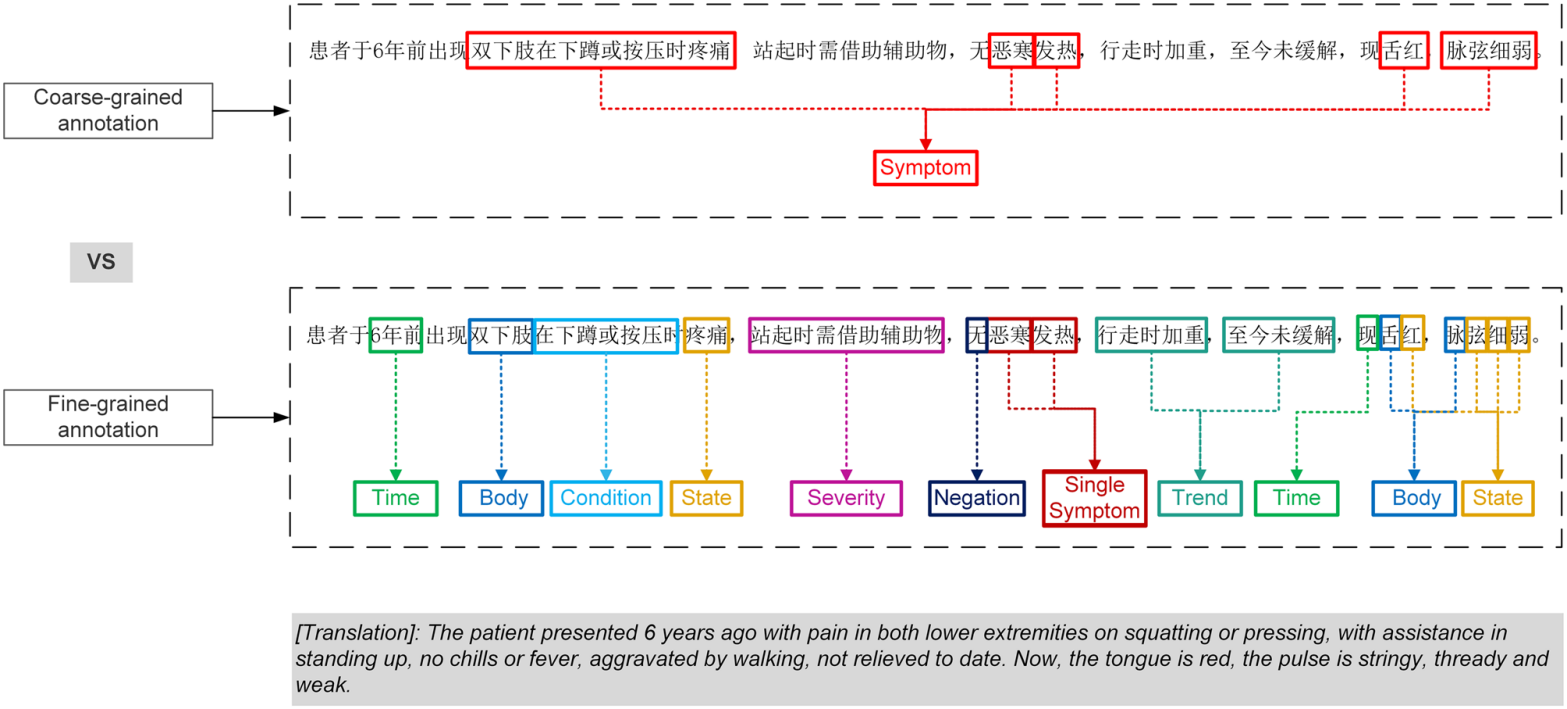
Example of coarse-grained vs. fine-grained annotation, translated into English for reference

Figure 1 displays the symptom information retrieved by the two approaches. First, the coarse-grained cannot distinguish the symptom “双下肢疼痛” (pain in both lower extremities) from the condition “在下蹲或按压时” (on squatting or pressing) and cannot split the compound symptom “脉弦细弱” (the pulse is stringy, thready and weak) into a single symptom “脉弦，脉细，脉弱” (Stringy pulse, thready pulse, weak pulse); second, the coarse-grained misses a lot of useful information, such as the time “6年前” (6 years ago) and the trend “至今未缓解” (not relieved to date), which can support the identification of “prolonged illness must be weak” and auxiliary treatment in TCM. Fine-grained extraction of the condition “在下蹲或按压时” (when squatting or pressing) and the trend “行走时加重” (aggravated by walking) can distinguish active pain from resting pain, and the severity of pain “站起时需辅助物” (with assistance in standing up) can further infer that the patient may have a change in a condition such as “yang deficiency” or “qi deficiency”. Most importantly, the negative word “无” (no) reveals negative symptoms for differential diagnosis and avoids completely opposite results. Therefore, coarse-grained extraction not only fails to solve the problem of symptom nesting but also has the problem of missing information, which makes it difficult to reflect the real condition of the patient fully and thus affects the accuracy and clinical efficacy of the TCM dialectic.

Some studies attempt to define these factors as attributes [12] and treat the relationships between entities as “relations” [13, 14]. However, natural language descriptions are highly diverse, and categorizing texts into attributes through manual analysis is subjective, often resulting in the loss of a large amount of original textual content and low information utilization. Zhang et al. [15] point out that SPANNER is more suitable for nested NER than sequence annotation. Based on SPANNER, they introduce the named entity head (NEH) prediction task, which significantly reduces computation and is suitable for datasets with more than 1,000 but fewer than 10,000 annotated sentences. Zhang et al. [16] also propose a span-based model to address the extraction of overlapping entities in the randomized controlled trial (RCT) literature. Their approach involves constructing entity relationships to identify overlapping entity structures. In addition, when the dataset is small, the effectiveness of relation-based recognition is limited [12], making it difficult to standardize and organize the data.

This study presents a fine-grained classification and normalized output system for TCM symptom information to overcome the limitations of existing methods and challenges. By syntactic analysis and fine-grained entity classification, combined with relation rules in grammatical structures, attributes and relationships are visualized as entities, clarifying the elements in symptom descriptions. This approach not only effectively reduces issues such as symptom nesting and discontinuity but also preserves the original text, improving information utilization. In addition, by normalizing the relationships between entities through the core concept of symptom, a systematic framework for describing symptoms is constructed, which improves data normalization. The study includes three tasks: fine-grained annotation guideline development, entity annotation, model training, and normalized output, with detailed descriptions of each process. The experimental data, consisting of 500 EMRs from a TCM hospital in Guangdong Province, are manually annotated and verified by experienced TCM doctors and graduate students. IAA F1 scores are used to assess annotation consistency. Additionally, the study evaluates the performance of several popular NER models on this dataset, with the best model achieving an F1 score of 92.29%.

The main contributions of this study are threefold:

1. We propose a medical semantic-based “position + state” symptom description structure and a fine-grained description framework for symptom information.
2. We develop a fine-grained annotation guideline for extracting symptom information from TCM EMRs (see Additional file 1). Three dimensions are defined, including 12 types of entities and 10 types of relations; the structure of symptom expression is further specified to ensure comprehensive representation.
3. The system utilizes relation combination rules to produce a normalized output. It provides an efficient and reliable technical guarantee for the standardized processing and precise analysis of symptom information and also lays a solid foundation for the construction and research of high-quality corpus.

## Related work

In recent years, processing for EMRs becomes an important branch in the development of medical intelligence, and NER and RE are two core tasks in the field of textual information extraction. Entity recognition is initially based on rules and dictionaries, which show obvious limitations in dealing with complex contexts, and machine learning models are difficult to fully adapt to the semantic richness in terms of feature engineering dependency [17]. Deep Learning (DL) models, on the other hand, are more mature and make significant progress by automatically learning contextual features. Nowadays, with the arrival of Large Language Models (LLMs) [18], such as ChatGPT [19], LLaMA [20], with their strong generative power and generalization performance, they bring new possibilities for tasks such as medical history data mining [21–24].

Large-scale and high-quality annotated datasets are among the most important foundations for successful machine learning modelling, especially for DL models. Currently, the main extracted medical information is based on top-level medical concepts defined in textbooks and national standards, such as symptoms, diseases, tests, treatments [25], and medications [26]. This approach helps to simplify complex clinical information systems, facilitates large-scale data integration and analysis, and provides rapid guidance for clinical practice and research [27].

Research on English EMRs starts earlier, and due to the specificity of participles in the writing of the language, there are now well-established medical information extraction studies and medical corpora [28–31]. The famous i2b2 (Informatics for Integrating Biology to the Bedside) project organizes a series of shared task challenges and publicly releases the corresponding corpora, based on which the research on medical information extraction in English makes significant progress. In comparison, medical information extraction research in China starts later, and the special characteristics of the Chinese language make it more challenging to develop. However, with the rapid development of AI and natural language processing (NLP) technology in recent years, relevant research in China gradually achieves some breakthrough results. For example, in 2017, the China Conference on Knowledge Graph and Semantic Computing (CCKS) starts to publish shared tasks for NER for Chinese EMRs, of which the 2019 version contains about 1,400 annotated documents covering diseases and diagnoses, anatomy, laboratory examination, imaging examination, medication, and surgery in 6 types of entities. Meanwhile, with the gradual deepening of medical information extraction research in China, more and more scholars begin to pay attention to the problem of entity recognition and information extraction in Chinese medical text. The main annotated corpora of Chinese medical information are summarized in Table 1.

**Table 1:** Studies on the construction of Chinese clinical text corpora in recent years

Author	Scale and target	Entities	RTC	FG
Wang Q [25]	1,596 annotated instances (10,024 sentences)	Diseases, symptoms, exams, treatments, and body parts	-	F
Gao et al.[13]	255 authentic admission records	Medical discovery, body parts, temporal words, diseases, medications, treatments, inspections, laboratory tests, and measurements	4	T
Cai et al. [32]	1,000 admission records	Anatomical parts, symptom descriptions, independent symptoms, drugs, and operations	-	F
Xiong et al.[33]	1,000 admission notes and 800 discharge summaries	Body parts, diseases, symptoms, tests, and treatments	-	F
Zhang et al.[5]	10,197 TCM clinical records	Ordinary body part, Tongue body, Tongue coating, Pulse, Acupoint, Meridian and collateral, Zang organ, Fu organ, Both the tongue body and tongue Coating, Tongue body manifestation, Tongue coating manifestation, Pulse condition, Direction and position	-	T
Zhang et al.[26]	4,000 medical records	Disease, Symptom, Test, Treatment, Medicine, Abnormal inspection result	-	F
Chang et al. [14]	41 articles on medical norms and consensus	Disease, Class, Reason, pathogenesis, Symptom, Test, Test_Items, Test_Value, Drug, Frequency, Amount, Method, Treatment, Operation, ADE, Anatomy, Level, Duration	15	T
Chen et al.[34]	1,000 EMRs	symptom, disease, exam, examination results, location	4	F
Cheng et al.[35]	736 discharge records	Anatomy, Operation, Drug, Independent symptoms, Describe symptoms	-	F
Lee et al.[36]	30,692 sentences	body, symptom, instrument, examination, chemical, disease, drug, supplement, treatment and time	-	T
Zou et al.[8]	48,191 Chinese EMRs texts	Presented symptom, Negated symptom, Disease, Tongue and pulse, Body parts, Operation, Date, Duration of symptoms, Past history, Inducement, Drug, Frequency, Principle	-	T
Zheng et al.[37]	Actual medical records (reaching 14,095 entities.)	Disease, Symptom, Age, History, Check, Method, Drug	-	F
Fang et al.[38]	Present illness, past medical history, case characteristics and family history of 500 pituitary adenoma inpatients	symptoms, body regions, diseases, family histories, surgeries, medications, and disease courses of pituitary adenomas	-	F
Wang et al.[7]	2,007 EMRs	Disease, Symptoms, Anatomy, Examination, Instrument, Medicine, Operation	-	F
Liu et al.[39]	1,986 medical Chinese EMRs	Disease, Symptom, Treatment, Physical examination, Imaging test, Lab test, Aggravating factor, Mitigation factor, Body part	11	T
Zhu et al.[12]	1,200 medical records	Disease (Disease or Syndrome, Injury or Poisoning, Organ Damage), Symptom (Self-Reported Abnormality, Abnormal Test Result), Test (Test Process, Test Result), Treatment (Treatment, Operation, Prevention, Care), Drug (Drug, Drug Dose), Body (Body Part, Body Matter), Personal History, Equipment, Department	10	T
Chu et al.[40]	1,000 Chinese EMRs	disease diagnosis, auxiliary examinations, symptom manifestations, treatment measures, and physical condition	-	F
Du et al.[41]	100 Chinese clinical EMRs	Diseases, Diagnoses, Imaging examinations, Lab tests, Surgeries, Medications, Anatomical sites	-	F

In the table “-” means no RE set. RTC: Relation Types Count; FG: Fine-grained; T: True; F: False

In order to unify the distinction between coarse-grained and fine-grained entities, this study defines that coarse-grained refers to treating symptoms, diseases, medications, treatments, and other items as basic units of the lowest dimension, only conducting entity recognition and extraction at the macroscopic level, without further decomposition or subdivision of the internal attributes, features, or associated information of various entities. In contrast, fine-grained refers to more detailed dimensional expansion and attribute division of the aforementioned basic units—for example, adding attribute dimensions such as “time”, “degree”, and “frequency”, or classifying and decomposing specific categories to which entities belong, such as further subdividing the macroscopic category of “body parts” into “tongue manifestations”, “pulse conditions”, and so on.

Based on coarse-grained dimensions, studies annotate diseases, symptoms, tests, treatments, and body parts [25, 33, 34, 40, 41]; after that, medication is further annotated [7, 26, 35, 37]; Cai et al. [32] distinguish independent symptoms from symptom descriptions; Zhu et al. [12] include body matter and body parts as subcategories of the body; Fang et al. [38] annotate the course of pituitary tumours. In addition, Gao et al. and Lee et al. incorporate the annotation of time vocabulary [13, 36]; Zou et al. and Chang et al. further extend the annotation of symptom duration and frequency [8, 14]; Liu et al. [39] annotate aggravating and mitigating factors.

For the characteristic contents of TCM, Zou et al. [8] further annotate tongue and pulse; Zhang et al. [5] annotate acupoints, meridian and collateral, Zang organ, and Fu organ in order to fully explore the special parts of TCM medical records, which helps to improve the utilization efficiency and research value of TCM clinical data. These studies lay an important foundation for intelligent processing and knowledge mining of medical texts, and also provide a reference for fine-grained studies.

Overall, although these annotation schemes are refined to a certain extent, one of the basic principles of TCM dialectics is holistic evaluation, and the above annotation schemes are still deficient in terms of comprehensiveness and meticulousness. ①The existing annotation schemes do not comprehensively take into account the symptom information in TCM medical records, resulting in the failure to effectively extract the tacit knowledge in the text, which limits the potential of the intelligent dialectic system to be applied in actual clinics, reduces the accuracy and reliability of the system, and also affects the interpretability of TCM dialectic; ②The annotated symptom information presents a certain degree of scattering, lacks of systematicity and structure, and low degree of correlation of the symptom information, which leads to insufficient reusability of the extracted knowledge, thus limiting its effective use in modernized technology.

## Methods

### Fine-grained TCM symptoms entities and relations normalized extraction process design

**Fig. 2:**
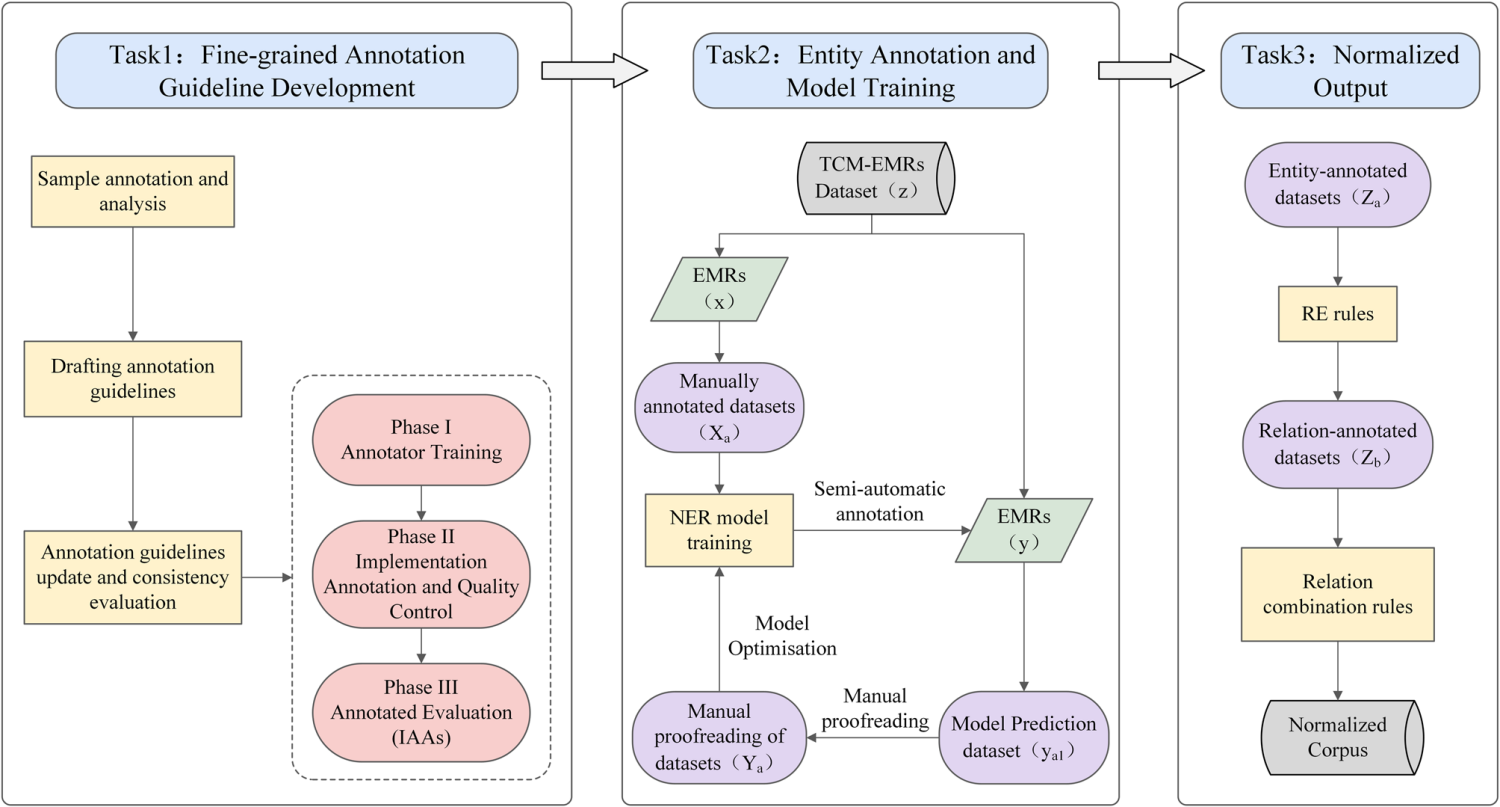
The workflow for the normalized output of symptom information (x, y, z denote the amount of unannotated data, (z = x+y); y_a1_ denotes the amount of data for the predicted entities using the NER model, X_a_, Y_a_, and Z_a_ denote the amount of data after the entities have been annotated and manually checked (Z_a_ = X_a_+Y_a_); and Z_b_ denotes the amount of data after the relations have been annotated and manually checked)

Based on the above, this study proposes a fine-grained annotation guideline and normalization output system for TCM EMRs, including the following three tasks, and Fig. 2 shows the whole workflow. The annotation team consists of two specialized doctors and two master’s degree students in TCM.

Task 1: Fine-grained annotation guideline development. (1) Sample annotation and analysis: Five medical records are randomly selected for sample annotation and analyzed to determine the types of entities and types of relations. (2) Drafting annotation guidelines: After referring to some well-developed research [5, 40, 42], we randomly select 50 records from the dataset; for entity annotation, the entity types are assigned into two groups, and two annotators independently annotate each group; for relation annotation, the records are divided into two groups, and each group is independently annotated by two annotators. By comparing the inconsistent annotation contents, after group discussion and reaching an agreement, a fine-grained annotation guideline is drafted. (3) Annotation guideline update and consistency evaluation: This process consists of three phases. The first phase organizes annotators to study the draft annotation guideline. In the second phase, during the implementation of annotation and quality control, through multiple rounds of iteration, the guidelines are continuously revised and improved based on feedback from annotators during the annotation process, ensuring they are more adaptable to the textual information in this study. The third phase is annotation evaluation, which follows previous studies and assesses the consistency of the annotated corpus through IAA.

Task 2: Entity annotation and model training. The entity annotation and NER model training for the entire dataset are completed in three phases of Task 1. Given the high cost of annotation, semi-automatic annotation is used in this study. That is, a simple model is trained on the annotated medical records and applied to the unannotated medical records. The annotators then proofread them, add them to the training set, and train a new model until all data are annotated and manually proofread, thereby reducing the workload of the annotators.

Task 3: Normalized output. Through the analysis of the dataset, we design the RE rules and combination rules, using the rules to extract the relations and manually check them, and then use the rules to combine the relations to achieve the normalized output of the entire dataset.

### Fine-grained annotation system

A study delineates the distinction between “symptoms and signs” and “clinical manifestations”, emphasizing that clinical manifestations encompass the temporal sequence and progression of symptoms, as well as their dynamic alterations in attributes such as colour, quality, quantity, and taste, which collectively represent the disease’s pattern of change [43]. Considering the attributes of TCM, this study collectively designates “symptoms” and “signs” as “symptoms” and encompasses “symptoms”, “signs”, and their associated ancillary information as “symptom information”.

This study examines symptom descriptions and identifies specific structural traits; the majority of symptoms may be categorized into “body parts” and “symptom elements” [43]. Nevertheless, these two concepts cannot comprehensively encompass all symptoms, so the article delineates this structure as “Position” and “State”. Further investigation reveals that the manifestation of symptom combinations exhibits a comparable amalgamation of root and affix within the English lexicon, which serves as a significant reference point for deconstructing the notion of “Position” in the detailed classification of this study into “Position_Scope”, “Position_Primary”, and “Position_Subordinate”.

#### Fine-grained entity and relation definitions

According to the existing definitions and research [8, 12, 43], combined with the need for accurate clinical identification and treatment in TCM, this study classifies TCM symptom information into 12 types of entities and sets 10 types of relations from three dimensions. The core focus of this study is on the integration of TCM symptoms and their collateral information. It does not cover either the superordinate or sibling concepts of the symptoms, such as “disease”, “drug”, “treatment”, nor does it include related content from Western medicine. Referring to the concept definitions of TCM in WHOs international standard terminologies on traditional medicine in the Western Pacific region [44] and a reference book on TCM symptom terminology [43], this study presents the definitions of 12 types of entities with examples in Table 2 with 10 relation types and their examples in Table 3. Since only relation types, not triad types, are set in the annotation tool, the same relation types are applied to the same object in this paper.

**Table 2:**
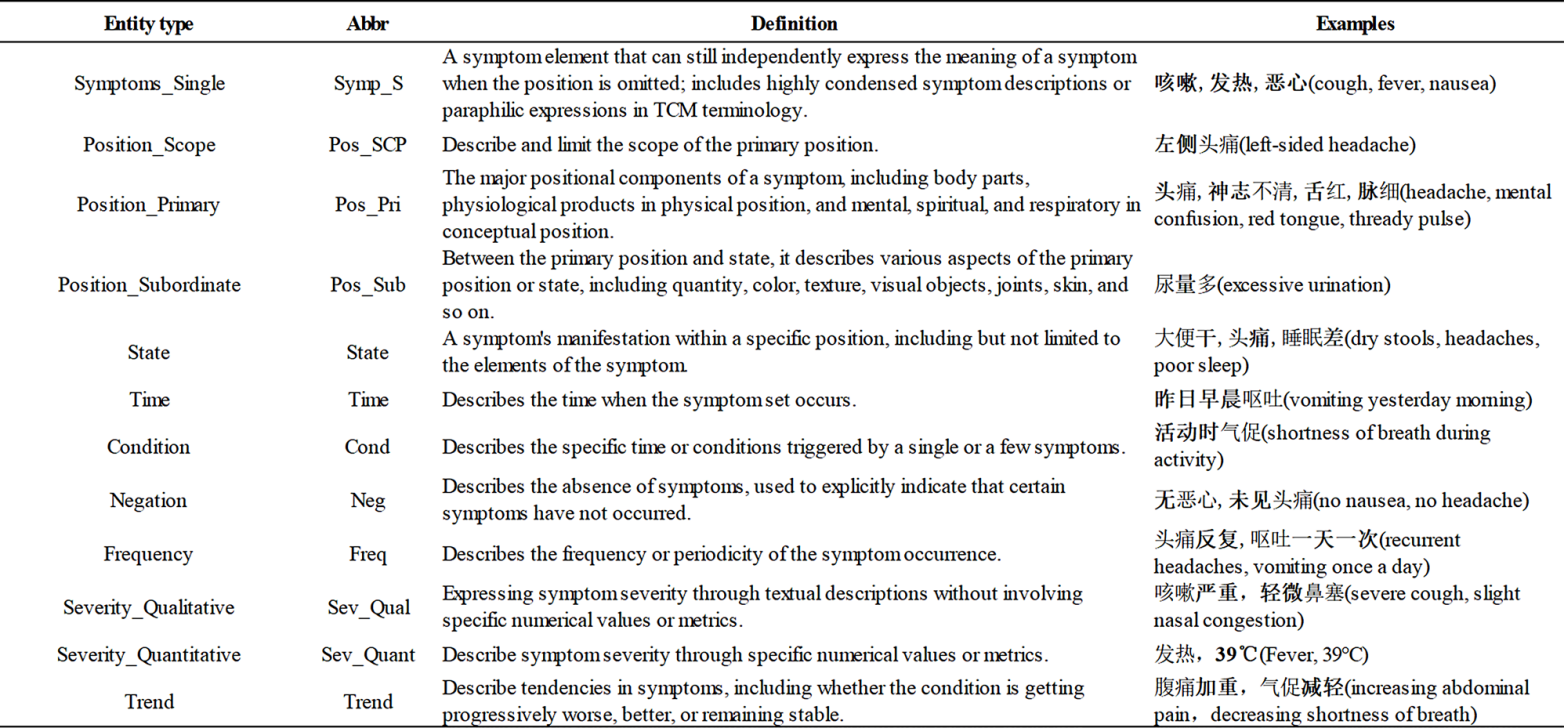
The definitions of the 12 types of entities in this study and their examples

Abbreviation. In the examples, the entities are in bold font

**Table 3:**
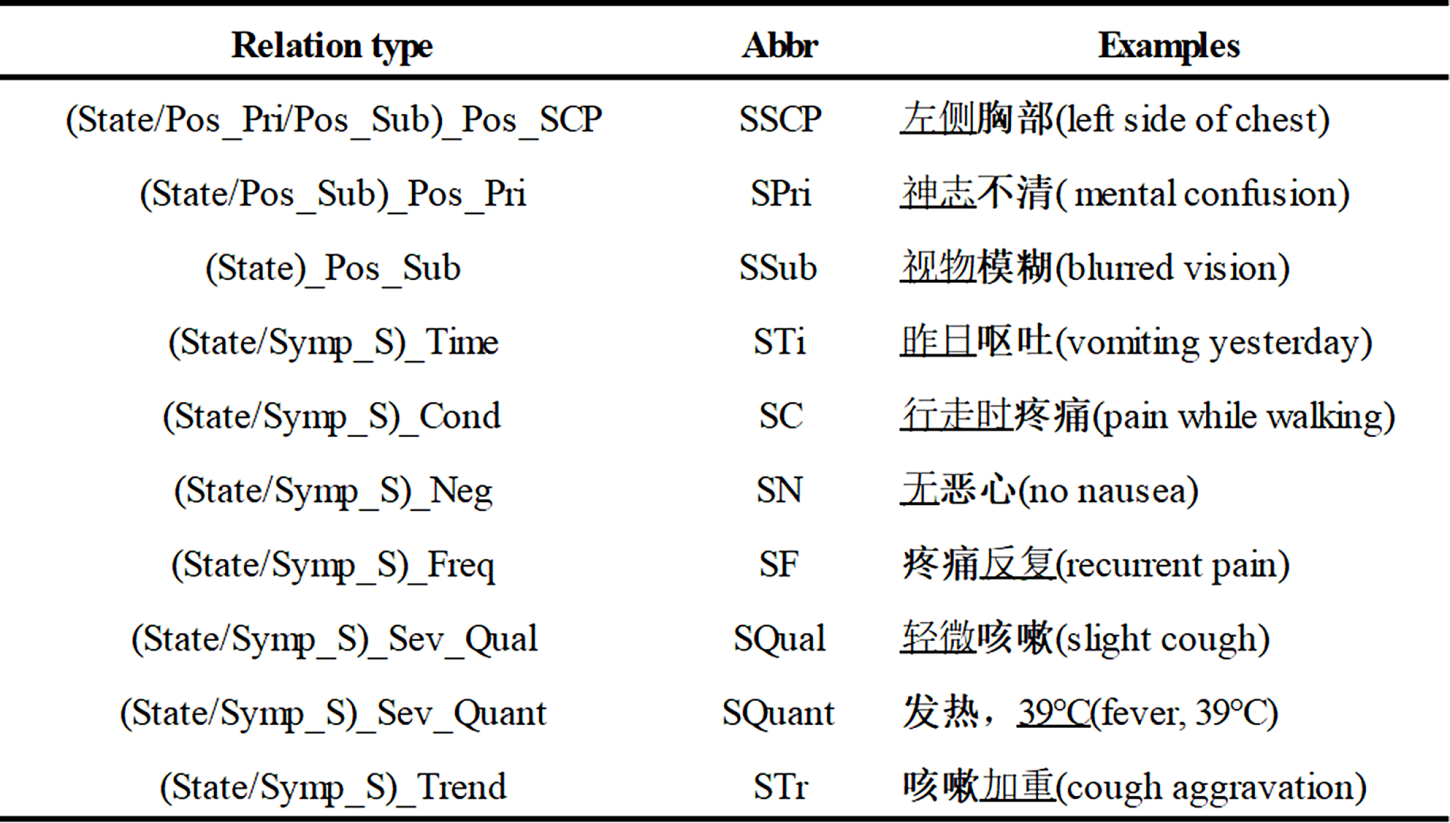
The 10 types of relations and their examples

In the examples, the subject is in bold and the object is underlined

The differences from the traditional classification of symptom information are as follows:

1. Symptom elements that can independently express the meaning of a symptom are defined as “Symptoms_Single”; the rest are defined as “States”. Symptom elements include, but are not limited to, the 19 categories of abnormality described in this book [43].
2. The subject of symptom occurrence is defined as “position”. It covers two categories, namely, physical position and conceptual position; physical position refers to the physical part that can be directly seen and touched, corresponding to anatomy, secretions, excretions; conceptual position refers to the intangible and abstract existence, corresponding to mental activities, life functions. According to the characteristics of positional description, “position” is further divided into three parts, including “Position_Scope”, “Position_Primary” and “Position_Subordinate”; the “Position_Primary” is explicitly defined as “anatomy part, organ, spirit, mental, consciousness, behaviour” and other categories as the first subject; the “Position_Scope” is the description and limitation of the range of the “Position_Primary”, such as the range of an area delineated by “left, right, bilateral, upper, lower, major, minor”; the “Position_Subordinate” entity, located between the “Position_Primary” and the “State”, describes various aspects of either the “Position_Primary” or the “State”, including quantity, colour, quality, visual objects, joints, skin. The lack of “Position_Subordinate” sometimes causes ambiguity in the presentation of clinical information, depending on the “State”; for different kinds of states, the actual content of the relevant “Position_Scope”, “Position_Primary”, “Position_Subordinate” is different, and they do not correspond on a one-to-one basis. We select the most appropriate type of entity based on the specific situation and its semantics.
3. The time that describes the occurrence of a set of symptoms is defined as “Time”, and the specific time when a single or several symptoms occur or a specific condition arises as a “Condition”.
4. Descriptions of symptom severity that rely on qualitative textual descriptions, without specific numerical values or metrics, are defined as “Severity_Qualitative.” In contrast, descriptions that quantify symptom severity using specific values, metrics, and other measurable criteria are defined as “Severity_Quantitative”.

#### Fine-grained description framework

**Fig. 3:**
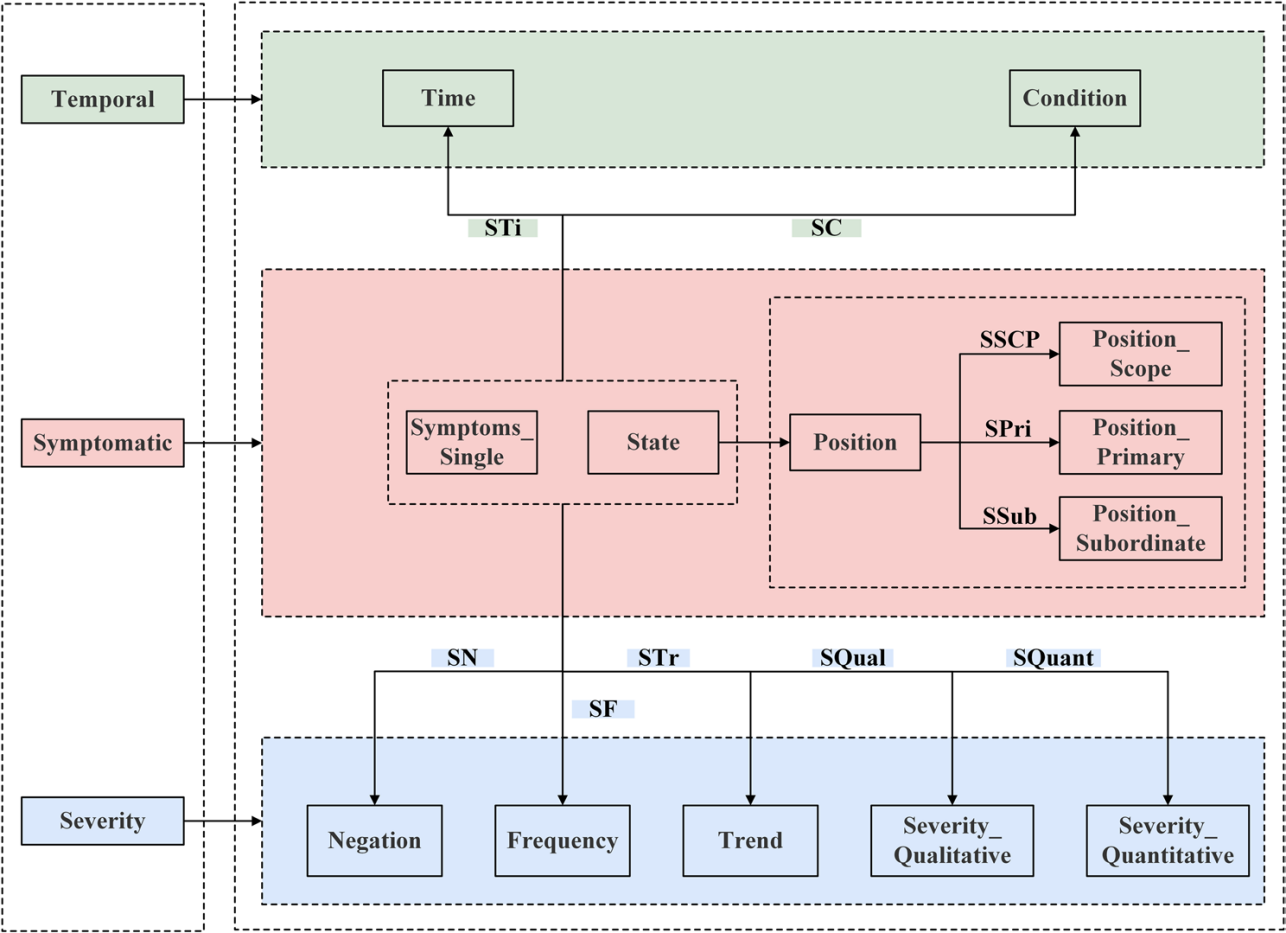
A framework for fine-grained description of symptom information

Based on the above definition, we construct a framework for fine-grained description of TCM symptom information, as shown in Fig. 3; and visualize the relations between 12 types of entities, through which the hierarchical structure and interconnections between symptom information can be demonstrated more clearly.

#### Data sources and processing

##### Data sources

**Fig. 4:**
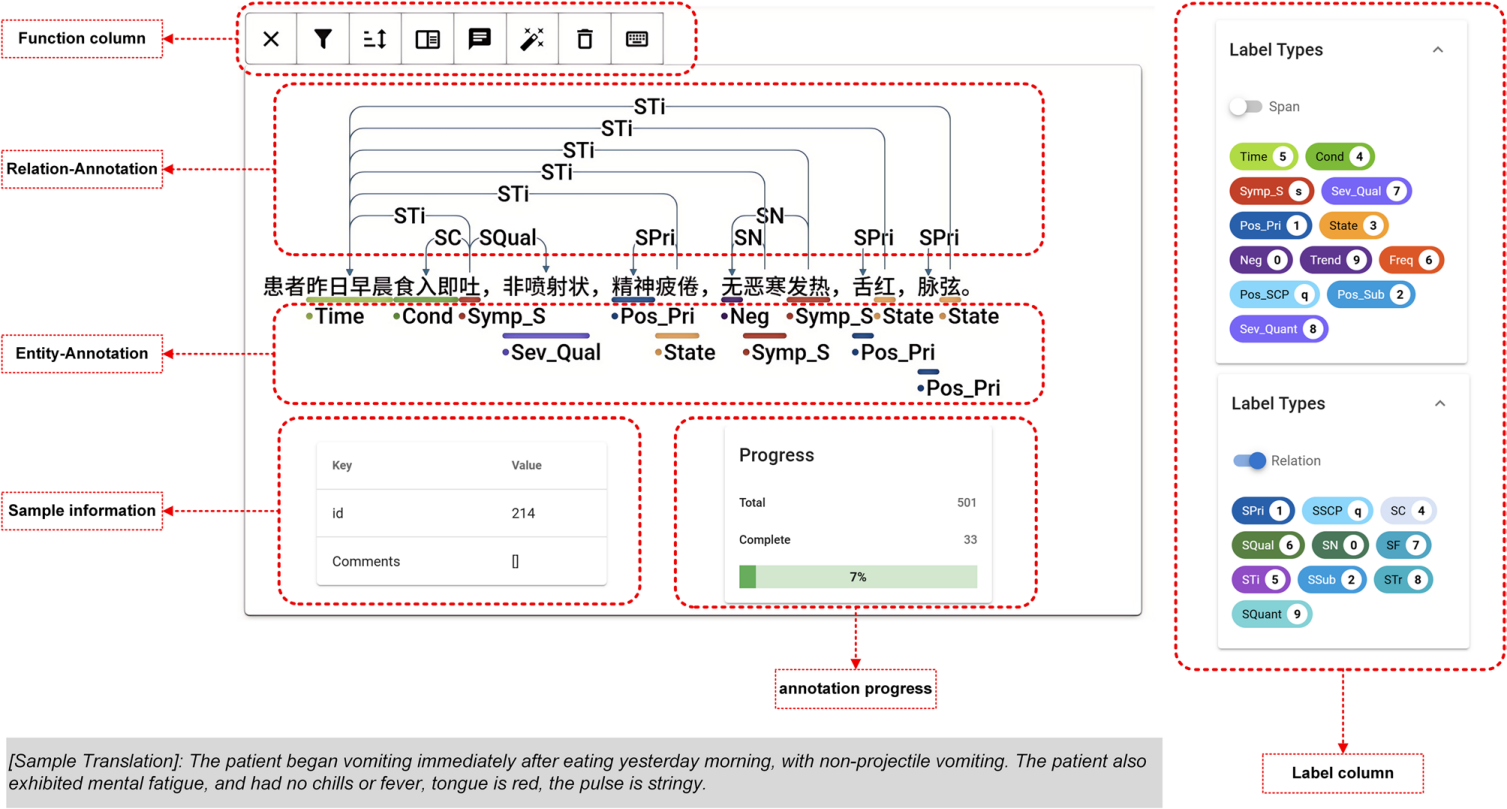
EMR sample and annotation interface, translated into English for reference

In this study, we select 500 EMRs from a TCM Hospital in Guangzhou City, Guangdong Province, China, as the database for the study (hereafter referred to as GDTCM-500). To ensure the relevance of the data and the effectiveness of the research, the present illness history section, which is most closely related to diagnosis and syndrome differentiation, is chosen as the focus of the study. The source and use of the data were reviewed by the Ethics Committee of the Guangdong Second TCM Hospital. The ethical approval number is Y202407-004–01. A data sample and annotation interface are shown in Fig. 4.

##### Data preprocessing

In order to ensure that the study is based on real-world medical texts and in conjunction with the goal of this study, which is to extract the structure of normalized symptom information expression, this study chooses not to perform cleaning and preprocessing operations on the raw data, except for the part related to privacy. This operation aims to better evaluate the robustness and adaptability of the proposed method in real-world application scenarios. Furthermore, the fine-grained entity classification scheme developed in this study effectively filters out noisy text while maintaining well-defined entity boundaries. This approach not only enhances entity recognition performance but also facilitates efficient data cleaning.

#### Annotation tool

After comparing various annotation tools, we finally choose the open-source Doccano [45], which realizes the annotation of entities and relations in the same text and enables collaborative annotation by multiple people, which improves the annotation efficiency. In addition, it also allows users to set different colours for the specified entities and relations so as to show the effect of annotation more obviously and enhances the visualization and accuracy of the annotation.

#### Annotation principles

##### Entity annotation principles

In addition to following the fine-grained annotation guidelines developed, this study also follows the following basic principles for entity annotation:

1. No overlapping annotation: an entity cannot be annotated as two or more entity types;
2. No nested annotation: the appropriate entity type is selected according to the contextual semantics when annotating, and an entity cannot be annotated again in an entity;
3. Maximize coverage annotation: entities that have progressive relationships within the same type should implement maximum coverage annotation. For example, in “左手小指疼痛” (pain in the little finger of the left hand), “手小指” (little finger of the hand) is the main position with a progressive relation, so the whole is annotated as the “Position_Primary”.

##### Relation annotation principles

Relations are relatively high-level semantics, so ideally, annotating symptom relations relies on both the correctness of the text and its consistency with external knowledge. However, real clinical texts contain a large number of non-negligible typos, grammatical errors, and content errors that make symptom relations difficult to interpret or lead to conflicts between textual meaning and external knowledge. As mentioned earlier, annotation should follow text literalness more preferentially than external knowledge margins. Thus, as long as the text clearly presents symptom-information relations, the annotator should annotate them accordingly; otherwise, the annotator may try to infer the implied relations from the text with the help of his medical knowledge. The following principles of relation annotation are followed:

1. “State” as subject: Except for the relation between “position” and “state”, the rest of the relations takes “state” as subject;
2. Progressive annotation: In the “position” relation annotation, follow the progressive order of annotation from “Position_Subordinate” → “Position_Primary” → “Position_Scope”.

#### Annotated evaluation

Inter-annotator agreements (IAAs) are metrics used to measure the consistency of different reviewers’ ratings or annotations of the same object or phenomenon and are often used to solve the problem of subjective judgments that cannot be observed through the senses. Especially in annotation or scoring tasks, the opinions of different reviewers may differ, so a metric is needed to determine the consistency among reviewers. In this study, we refer to the evaluation model of Zhu et al. [12] A certain number of EMRs are distributed to different annotators, and the result of one annotator is taken as the true value, and the result of the other annotator is taken as the predicted value. Then, their consistency is compared.

1 $${\text{R}} = \frac{{{{\left| {\text{y}} \right.}^1} \cap \left. {{{\text{y}}^2}} \right|}}{{\left| {{{\text{y}}^1}} \right|}}$$

2 $${\text{P}} = \frac{{{{\left| {\text{y}} \right.}^1} \cap \left. {{{\text{y}}^2}} \right|}}{{\left| {{{\text{y}}^2}} \right|}}$$

3 $${{\text{F}}_1} = \frac{{2 \times {\text{P}} \times {\text{R}}}}{{{\text{P}} + {\text{R}}}}$$

Where y^1^ and y^2^ are the annotations of two annotators, respectively, where y^1^ stands for “real annotation” and y^2^ stands for “predictive annotation”. Precision (P): the proportion of annotations where y^2^ agrees with y^1^ to y^2^. Recall(R): the proportion of annotations where y^2^ agrees with y^1^ to y^1^. F_1_ score: the average of the sum of P and R. It combined P and R are considered. When y^1^ and y^2^ are exchanged, the F_1_ score remains unchanged.

### DL-based symptom information NER experiment

In order to achieve a normalized extraction of automated entities and relations, common and well-established model modelling techniques are selected in this study, not only to validate the effectiveness of our fine-grained symptomatic information classification corpus but also to provide a baseline for future research.

#### Model structure

##### Long short-term memory network (LSTM)

The LSTM model [46] effectively filters and retains input sequences by introducing a gating mechanism (including input gates, output gates, and forget gates), thereby enabling long-term memory of specific information. This mechanism effectively alleviates common issues in traditional recognition tasks, such as gradient vanishing and gradient explosion, significantly improving the model’s stability and performance when processing sequential data [47, 48]. By adding a bidirectional LSTM (BiLSTM), it can simultaneously capture both forward and backward context information, further enhancing the model’s understanding and prediction ability of global semantics, making it one of the mainstream choices for entity recognition tasks in the field of TCM.

##### Transformer-based bi-directional encoder

In 2018, Google introduced Bidirectional Encoder Representations from Transformers (BERT) [49], which significantly improves the performance of NLP tasks by pretraining on large-scale unlabelled corpora and combining bidirectional context modelling (Masked Language Model, MLM, and Next Sentence Prediction, NSP) to learn deep semantic representations of text. As the technology evolves, RoBERTa is derived by removing the NSP task from BERT, increasing the training data volume, extending the training time, and adjusting training parameters to improve BERT’s performance. Additionally, by modifying the Whole Word Masking (WWM) strategy, BERT-wwm and RoBERTa-wwm [50] introduce to address the issue of handling individual characters or sub-words during masking in BERT, thus enabling the model to better understand Chinese and improving the comprehension of Chinese text.

##### Conditional random fields (CRF)

CRF [51] was proposed by Lafferty in 2001. By modelling the global dependencies of the label sequence, it ensures the structural consistency of the predicted results. When combined with neural networks, it effectively utilizes contextual features and optimizes the prediction of label sequences, thereby improving the accuracy and robustness of sequence annotation tasks.

#### Experimental settings

In this study, experiments are conducted on GDTCM-500. It is divided into a training set and a test set, including 400 and 100 annotated TCM EMRs, respectively. The training set is used for model training, and the test set is used for performance evaluation. The evaluation metrics, i.e., P, R, and F1 score, are computed in the same way as in previous IAAs evaluations, and the experiments use a strict evaluation criterion: an entity or relation is judged to be correctly predicted when and only when all of its constituent elements (including boundaries and types) are correct.

The model uses a character-based BiLSTM or a pre-trained language model as the embedding layer or encoder structure and combines it with a CRF layer to further optimize the sequence annotation results by modelling the dependencies between the labels. The character-based BiLSTM is trained from the beginning with a character embedding dimension of 100, random initialization, and the hidden size of 256. To prevent overfitting, a dropout rate of 0.5 is applied, with a learning rate of 1e-3, and a batch size of 100 for 70 epochs. The number of training rounds is determined according to the performance of the loss-value curve to ensure that the model can achieve better results in sufficient training time, and the threshold for gradient clipping is set to 5.0; for the Chinese pre-trained language model, BERT-Base-Chinese [49] and Chinese-RoBERTa-wwm-ext [50] are used, respectively, with a learning rate of 3e −5, batch size of 10 for 50 epochs, max sequence length 256, and the AdamW optimizer is configured with an epsilon value of 1e-8.

### Rule-based symptom information RE experiment

In this study, to address the characteristics of fine entity delineation of symptom information and complex semantic association in TCM medical record text, the joint extraction model Casrel [53] is first used to conduct experiments on the GDTCM-500 dataset. However, the experimental results show that the F1 value of the model only reaches 23.07%, which fails to achieve the expected results. To determine whether the observed limitations stem from modelling constraints, we evaluate our joint model on the publicly available DuIE2.0 dataset [54]. The experimental results demonstrate robust performance, achieving an F1 score of 70.69%, which confirms the model’s accuracy and generalization capability. Following comprehensive analysis, we identify several key limiting factors: first, the present medical history text has significant long text characteristics (the average length of a single text paragraph reaches 300–500 characters) and contains a high density of entity relations (a single text contains more than 80 sets of entity relation pairs), which makes it difficult for the model to efficiently capture the deep semantic association features with a limited size of the training samples (*n* = 500); second, the validation through annotation finds that there are explicit syntactic features and logical association rules between the symptomatic information entities, and this method has been shown to achieve the required experimental results; based on this, the construction rules for the RE experiments are finally determined.

#### Rules for RE

By analysing the annotated content in the text of medical records, it is found that there is a strong regularity in the fine-grained symptom information relations constructed in this study, which adopts a rule-based RE experiment and summarizes the following five RE rules:

1. Identify “State” as the subject, and search for the left and right neighbouring entities:

① If both the left and right neighbours are “Pos_Pri”, then it generates an “SPri” relation with the left one;

② If the right side is “Pos_Pri”, and the left side is not, then generate an “SPri” relation with the right side;

③ In other cases, find the first “Position” entity to the left; if it is “Pos_Pri”, generate “SPri” relation; if it is “Pos_Sub”, generate “SSub” relation; if it is “Pos_SCP”, generate “SSCP” relation.

(2) Identify “Pos_SCP” as the object, and look for the first neighbouring “Position” entity to the right. Whether it is “Pos_Pri” or “Pos_Sub”, it generates an “SSCP” relation with “Pos_SCP” as the object.
(3) Identify “Neg”, then recognize all “State” or “Symp_S” entities to the right. Stop the recognition upon encountering a “，” or “。” and generate the relation “SN” with the “Neg” as the object and “State” or “Symp_S” as the subject.
(4) Each “Time” entity, before encountering the next “Time”, is treated as the object and generates the “STi” relation with every “state” and “Symp_S” within the range as the subject.
(5) Identify “Cond”, “Trend”, “Freq”, “Sev_Qual”, and “Sev_Quant” as the object. Based on “，” and “。” for segmentation, generate corresponding relations “SC”, “STr”, “SF”, “SQual”, and “SQuant” with all “State” or “Symp_S” in the respective segmented sentence.

### Rule-based normalized corpus construction experiment

Building upon our fine-grained entity and relation annotation framework, this study introduces a novel set of relation combination rules. These rules systematically address three persistent challenges in clinical text processing: (1) symptom nesting, (2) symptom discontinuity, and (3) incomplete symptom information association. Through this methodology, we achieve standardized, normalized outputs suitable for downstream analytical applications. The specific rule formulations are as follows:

Rule 1: Combination of “Position” and “State.” Based on the recursive relation where “State” points to “Position”, starting from the current “State” entity, recursively search for the related “Position” node paths (including “Pos_Pri”, “Pos_Sub”, and “Pos_SCP”). During the path construction, update the label and corresponding text content of the current entity step by step into the path and recursively traverse the “Position” relations. If no further “Position” relations are available, record the current path and return. Different paths correspond to different output lines. To prevent circular references during recursion, a “visited” set is used to mark the entities that have been visited.

Rule 2: Combination of “State” and other relations. Traverse all other relations (such as “STi” or “SC”) with the same “State” or “Symp_S” entity as the subject. Classify the related text content according to the type of relation and store it in different attribute lists. If there is more than one of the same types of relation, they are connected using a “;”.

Rule 3: Complete information combination. Combine the output of the same “State” or “Symp_S” entity after applying rule 1 with the output of rule 2, and output it to the corresponding column positions to form a complete structure of symptom information.

## Results

### Annotation results

For 500 TCM EMRs, the final total number of entities annotated is 39,097. The results of the annotation of various entities are shown in Table 4. The total number of annotated relations is 41,373, and the results of each type of relation annotation are shown in Table 5.

**Table 4:** Entity annotation results

Entity Type	Label	Entity description count	Shortest Character Length	Longest Character Length	Annotation Count	Annotation Percentage
State	State	823	1	9	12584	32.20%
Position_Primary	Pos_Pri	363	1	9	11482	29.40%
Negation	Neg	10	1	3	3097	7.90%
Symptoms_Single	Symp_S	212	1	6	2770	7.10%
Position_Scope	Pos_SCP	52	1	4	2230	5.70%
Time	Time	623	1	21	1991	5.10%
Position_Subordinate	Pos_Sub	95	1	16	1076	2.80%
Severity_Qualitative	Sev_Qual	277	1	24	1043	2.70%
Frequency	Freq	221	1	28	988	2.50%
Condition	Cond	239	1	19	893	2.30%
Trend	Trend	507	1	141	719	1.80%
Severity_Quantitative	Sev_Quant	136	1	28	224	0.60%
Total	-	-	-	-	39097	100.00%

**Table 5:** Relation annotation results

Relation Type	Label	Annotation Count	Annotation Percentage
(State/Symp_S)_Time	STi	15574	37.60%
(State/Pos_Sub)_Pos_Pri	SPri	12361	29.90%
(State/Symp_S)_Neg	SN	5185	12.50%
(State/Pos_Pri/Pos_Sub)_Pos_SCP	SSCP	2295	5.50%
(State/Symp_S)_Cond	SC	1564	3.80%
(State/Symp_S)_Freq	SF	1199	2.90%
(State)_Pos_Sub	SSub	1169	2.80%
(State/Symp_S)_Sev_Qual	SQual	1156	2.80%
(State/Symp_S)_Trend	STr	640	1.50%
(State/Symp_S)_Sev_Quant	SQuant	230	0.60%
Total	-	41373	100.00%

In order to verify the annotation quality, we randomly select 50 medical records and distribute them to different annotators and collect and compare the annotation results on the same records by calculating the IAAs. The IAAs for entity and relation annotation are 93.56% and 91.23%, respectively.

Due to the large difference in the distribution of entity type counts, with “State” (37.6%) accounting for the largest proportion and “Severity_Quantitative” (0.6%) accounting for the smallest proportion, we conduct the following analysis: Firstly, symptoms are the most important information in TCM EMRs. Among all symptoms (39.3%), “Symptoms_Single” accounts for 1/5, and the rest of the symptoms are composed of “State”, so the proportion of “State” is the largest. Secondly, each “State” entity is associated with a “Position”, but due to semantic differences, the three “Position” entities are omitted to varying degrees depending on the situation. When “Position_Subordinate” is omitted, it usually does not affect the expression of the symptom. For example, “双眼视物模糊” (blurred vision in both eyes) can be simplified as “双眼模糊” (both eyes are blurry), and “痰质稠” (thick phlegm consistency) can be simplified as “痰稠” (thick phlegm), and this simplification basically does not affect the understanding of the symptom. Therefore, when describing the symptoms, doctors tend to ignore the description of the “Position_Subordinate”. However, omitting the “Position_Primary” can lead to ambiguity in the symptom. For example, if the word “口” (mouth) is omitted from “口干” (dry mouth), it is not possible to determine whether it describes “口干” (dry mouth), “大便干” (dry stool) or “全身皮肤干” (dry skin all over the body). Therefore, the “Position_Primary” usually appears together with the “State”, but when the “Position_Primary” of multiple “States” is the same, it may be described only once; in addition, some symptoms may involve a specific scope, which is described together with the “Position_Primary”. Thus, in the “Position” section, the proportion of “Position_Primary” entities is the largest (29.4%), followed by “Position_Scope” entities (5.7%), and finally, “Position_Subordinate” entities (2.8%). In addition, strictly speaking, any symptom should describe its severity. However, due to factors such as the patient’s vague memory or the doctor’s assessment that it is temporarily unimportant, severity descriptions are often lacking. Quantitative descriptions of severity usually rely on professional tools (such as thermometers, measuring cups) for measurement, and not all symptoms can be quantified in terms of severity. For example, symptoms like “chills” or “nausea” are difficult to quantify in terms of severity, which is why the proportion of “Severity_Quantitative” entities is the smallest (0.6%).

#### Practicality comparison for TCM symptom information

In order to further validate the practicality of fine-grained annotation of symptom information in this study, we compare it with domestic and international studies that focus on fine-grained annotation in the following four aspects: “label for TCM symptom information annotation (TCM-SIA)”, “effectively mitigates TCM symptom nesting issue (TCM-SNI)”, “TCM-SIU” and “Normalized symptom information output (NSIO)”. The results are shown in Table 6.

**Table 6:** Comparison of practicality applied to TCM symptom information

Author	Label for TCM-SIA (count)	Mitigate TCM-SNI	TCM-SIU	NSIO
Gao et al. [13]	Medical discovery, Body parts, Temporal words（3）	T	45.33%	F
Zhang et al. [5]	Ordinary body part, Tongue body, Tongue coating, Pulse, Acupoint, Meridian and collateral, Zang organ, Fu organ, Both the tongue body and tongue Coating, Tongue body manifestation, Tongue coating manifestation, Pulse condition, Direction and position（13）	F	20.21%	F
Chang et al. [14]	Reason, Symptom, Frequency, Anatomy, Level, Duration（7）	F	62.43%	F
Lee et al. [36]	Body, Symptom, Time（3）	F	50.86%	F
Zou et al. [8]	Presented symptom, Negated symptom, Tongue and pulse, Body parts, Date, Duration of symptoms, Past history, Inducement, Frequency（9）	F	59.64%	F
Liu et al. [39]	Symptom, Aggravating factor, Mitigation factor, Body part（4）	T	42.01%	F
Zhu et al. [12]	Symptom(Self-Reported Abnormality), Body(Body Part, Body Matter), Attribute type(Negation, Uncertainty, Conditionality, Occasionality, Better, Worse, History)（10）	T	62.52%	F
Ours	Symptoms_Single, Position_Scope, Position_Primary, Position_Subordinate, State, Time, Condition, Negation, Frequency, Severity_Qualitative, Severity_Quantitative, Trend（12）	T	70.76%	T

We randomly select 10 medical records from the dataset and segment them into sentence units, resulting in 43 statements − 33 of which contain TCM-related content. Following the entity definitions and annotation examples provided by various researchers in their publications, our team manually annotates these 33 statements and calculates the annotation rate for each sentence (defined as the proportion of annotated characters to the total characters). The average annotation rate across all 33 TCM sentences serves as the primary metric for evaluating overall annotation effectiveness. Notably, when entity concepts from prior studies align with those defined in this paper, we directly adopt the existing annotations to avoid redundant labelling efforts.

Our comparative analysis reveals that the fine-grained classification system developed in this study offers substantial advantages for processing TCM medical records. The framework significantly enhances symptom information annotation through the systematic incorporation of six critical entity types: “Condition”, “Negation”, “Frequency”, “Severity_Qualitative”, “Severity_Quantitative”, and “Trend”. This comprehensive taxonomy yields measurable improvements, demonstrating at least an 8.24% increase in TCM-SIU metrics compared to conventional approaches. More importantly, our methodology successfully addresses two persistent challenges in clinical text analysis through its innovative integration of relationship annotation protocols and rule-based combination techniques. By simultaneously resolving issues of symptom nesting and information discontinuity inherent in coarse-grained annotation systems, the proposed approach generates structured outputs that serve multiple critical functions. These standardized data representations not only provide reliable inputs for intelligent diagnostic systems but also facilitate clinical decision-making processes. Ultimately, this advancement contributes significantly to ongoing efforts to modernize and standardize TCM diagnostic practices, bridging the gap between traditional medical knowledge and contemporary computational analysis requirements.

### NER experimental results

The comparison results of different models on GDTCM-500 NER are shown in Table 7. In addition, the experimental results of this study are compared with the articles with the closest TCM-SIU, and the comparison results are shown in Table 7. In the HwaMei-500 study, entity recognition and attribute recognition are divided into two sub-tasks, and here the average value is taken for comparison.

**Table 7:** Recognition results of different ner models

Model	GDTCM-500			**HwaMei-500** [12]		
	P	R	F1	P	R	F1
BiLSTM+CRF	**92.23%**	92.21%	92.22%	87.93%	87.44%	87.67%
BERT-Base-Chinese+CRF	90.07%	93.89%	91.94%	88.86%	89.69%	89.28%
BERT-Base-Chinese+BiLSTM+CRF	90.47%	**94.12%**	92.26%	89.06%	90.01%	89.53%
Chinese-RoBERTa-wwm-ext+CRF	90.37%	93.72%	92.01%	89.08%	90.10%	89.59%
Chinese-RoBERTa-wwm-ext+BiLSTM+CRF	90.73%	93.90%	**92.29%**	**89.31%**	**90.43%**	**89.87%**

The best experiment results are in bold

According to the experimental results, it can be seen that a simple character-based BiLSTM already obtains relatively high F1 scores, and the pre-trained language models achieve very similar performance, with a gap of no more than 1% in the F1 scores, and the optimal model reaches 92.29%, which indicates that the dataset constructed by the proposed fine-grained entity classification is of high quality, with a clear boundary of entities. Based on the comparison of the study, it can be seen that the fine-grained entity classification designed in this study has the best F1 result under the same model, which is improved by 2.43%. The entity recognition results for each entity type are also shown in Table 8.

**Table 8:** Recognition results for each entity type

Entity Type	Label	P	R	F1
Position_Primary	Pos_Pri	95.02%	96.13%	95.57%
Position_Scope	Pos_SCP	96.80%	96.19%	96.49%
Position_Subordinate	Pos_Sub	85.29%	84.47%	84.88%
State	State	92.30%	93.62%	92.95%
Symptoms_Single	Symp_S	93.39%	92.31%	92.84%
Condition	Cond	85.71%	82.42%	84.03%
Time	Time	92.00%	88.25%	90.09%
Frequency	Freq	81.73%	80.57%	81.15%
Negation	Neg	98.26%	97.75%	98.01%
Severity_Qualitative	Sev_Qual	75.27%	67.63%	71.25%
Severity_Quantitative	Sev_Quant	89.19%	82.50%	85.71%
Trend	Trend	54.35%	56.39%	55.35%

We compare the above results with those in Table 4 and identify the following phenomena:

1. The semantic complexity of entities is negatively correlated with recognition performance, meaning that simpler entities are easier to recognize. The fewer the entity description types, the higher the recognition performance. For example, comparing “Negation” (with 10 entity description counts and an F1 score of 98.01%) with “Symptoms_Single” (with 212 entity description counts and an F1 score of 92.84%) clearly illustrates this point.
2. Fluctuations in the description text length are a key factor in performance degradation. The higher the consistency in length, the more robust the model’s boundary judgment. For example, comparing “Trend” (with entity length fluctuation ranging from 1 to 141 and an F1 score of 55.35%) with “Time” (with entity length fluctuation ranging from 1 to 21 and an F1 score of 90.09%), both of which have similar sample sizes, reveals the impact of length consistency on performance.
3. The number of entity samples has a moderating effect on performance. When entity semantic complexity and text length fluctuations are similar, the greater the number of entities, the better the recognition performance. For instance, comparing “State” (with 12,584 counts and an F1 score of 92.95%) with “Time” (with 2,230 counts and an F1 score of 90.09%) demonstrates this phenomenon.
4. Multivariate interactions reveal paths to performance optimization. The semantic complexity of entities and fluctuations in text length are the primary factors that affect the model’s recognition performance. For example, “Negation” (with 10 entity description counts and entity fluctuation ranging from 1 to 3) achieves peak performance with an F1 score of 98.01%. However, “Trend” (with 507 entity description counts and entity fluctuation ranging from 1 to 141) results in a significant performance drop, with an F1 score of only 55.35%.

The above phenomena also highlight the necessity of fine-grained entity classification. Due to the characteristics of the Chinese language, which lacks explicit word segmentation boundaries, Chinese entity recognition faces numerous challenges. Chen et al. [52] also emphasize that entity boundaries and entity length are critical factors influencing recognition performance. Therefore, the fine-grained classification designed in this study effectively reduces the diversity of descriptions within the same category through semantic constraints, thereby reducing the decision-making dimensions of the model and improving recognition performance.

### RE experimental results

We conduct an RE experiment on GDTCM-500, and the experimental results are shown in Table 9, which can achieve an 88.17% F1 Score by using the RE rules. This experimental result verifies the validity of the rule method on specific relation types; for example, the F1 of the four types of relations, namely, “SPri”, “SSCP”, “STi”, and “SN”, all reach more than 85%, which proves that the rules in the annotation specification of the clear scenarios are reliable; the “STr” with the lowest F1 score, whose regularity in the text is not strong and small in number, can be corrected in the manual proofreading phase to ensure the accuracy of the final normalized output.

**Table 9:** Results of re experiments

Relation Type	Label	P	R	F1
(State/Pos_Sub)_Pos_Pri	SPri	90.62%	83.77%	87.06%
(State/Pos_Pri/Pos_Sub)_Pos_SCP	SSCP	94.46%	93.46%	93.96%
(State/Symp_S)_Time	STi	90.64%	88.86%	89.74%
(State/Symp_S)_Neg	SN	93.47%	97.44%	95.41%
(State/Symp_S)_Cond	SC	94.31%	72.20%	81.79%
(State/Symp_S)_Sev_Qual	SQual	83.02%	68.24%	74.91%
(State/Symp_S)_Freq	SF	88.54%	76.96%	82.35%
(State)_Pos_Sub	SSub	85.09%	71.93%	77.96%
(State/Symp_S)_Trend	STr	74.35%	31.35%	44.10%
(State/Symp_S)_Sev_Quant	SQuant	92.22%	67.84%	78.17%
micro_avg		90.87%	85.63%	88.17%

### Normalized corpus construction results

**Fig. 5:**
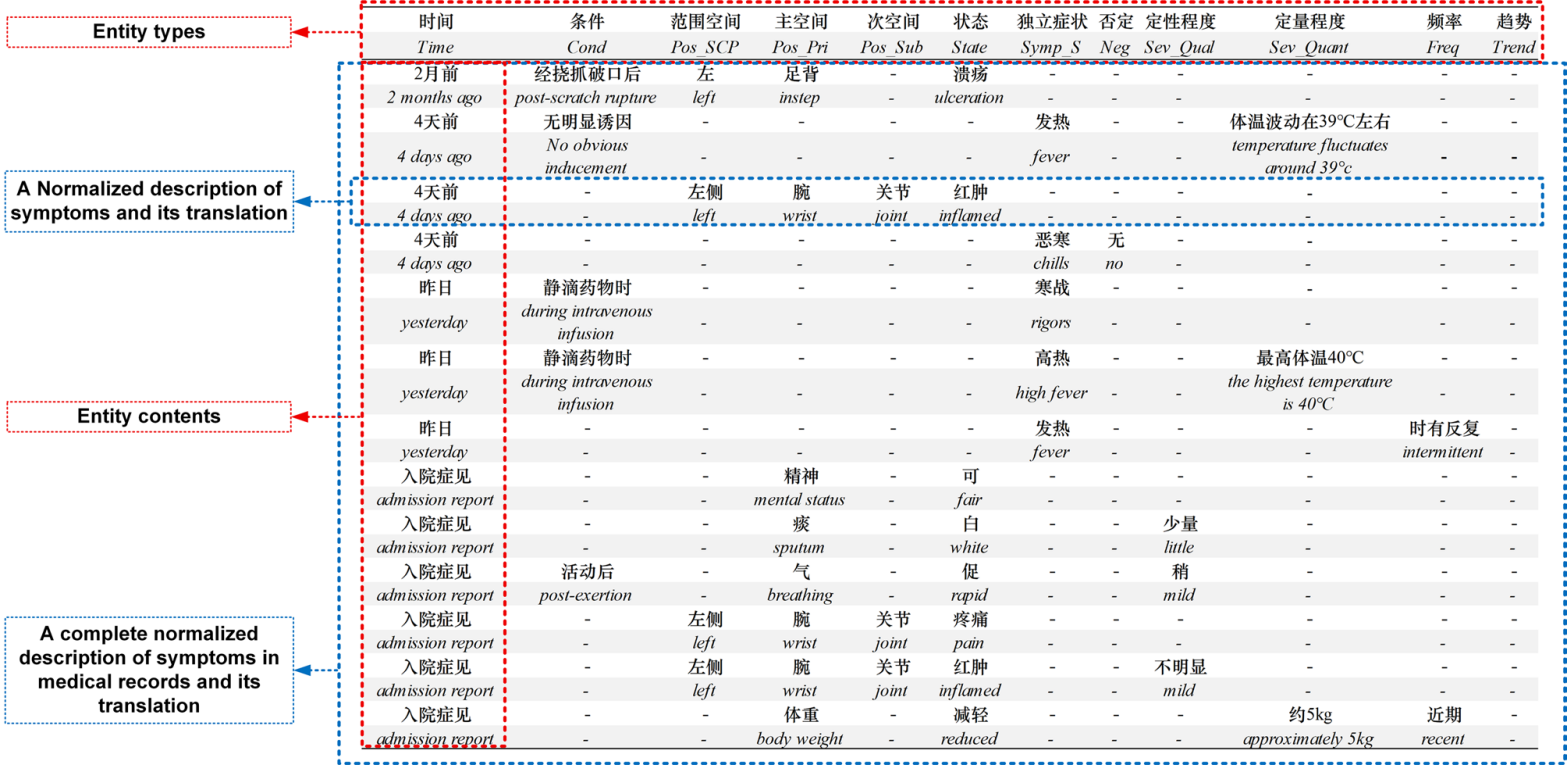
Example of normalized output, translated into English for reference (note: “-” in the figure indicates that no relevant information exists)

In this study, through fine-grained symptom information extraction, a standardized combined symptom information description framework is established by using entity relations to more comprehensively describe and express the core symptom information in TCM EMR. Using symptom information combination rules, each piece of scattered symptom information is organically combined with the “state” as the centre. As shown in Fig. 5, our system generates fully normalized clinical records where each line represents a standardized symptom statement. Complete records consist of multiple such statements, demonstrating our method’s capacity for structured clinical data representation. A total of 15,853 normalized symptom description statements are outputted for the GDTCM-500.

Through this framework, the complex clinical information in the medical record is effectively refined and structured to express, which lays the data foundation for subsequent knowledge mining, automatic diagnosis, and intelligent analysis of TCM. This combined way of describing clinical events not only reflects the characteristics of TCM evidence-based treatment but also enhances the applicability and interpretability of TCM medical records in multiple scenarios.

## Discussion

In the era of DL, algorithmic research is a hotspot in NLP, but the growing trend of model performance is gradually saturated in recent years. In contrast, the contribution of data is largely underestimated, as can be seen from the analysis of this study, the existing coarse-grained symptom classification [25, 26, 33] fails to effectively utilize the data information, and the existing fine-grained classification studies [8, 13, 36] are not comprehensive enough to fully integrate the relevant content of symptom information, reducing the performance of intelligent TCM dialectics.

In this study, we discover specific phenomena in symptom descriptions by analysing the annotated data. For example, modern TCM symptom descriptions often have similar grammatical structures and combination patterns of English root words and affixes, which provide important clues for symptom-normalized output. Based on these grammatical structures and combination patterns, we propose a symptom description structure based on the medical semantics of “position + state”, construct symptom information, and construct a fine-grained description framework for symptom information. At the same time, we develop a fine-grained annotation guideline for extracting symptom information from TCM EMRs. Through fine structural delineation, aspects of symptom information can be captured more accurately. The main purpose of this study is to output a normalized symptom description structure, which, combined with experimental results and manual proofreading, finally achieves a complete normalized output of symptom information in semi-structured EMRs.

### Limitations

This study had some limitations. First, the current EMR dataset’s single-source origin and limited scale constrain sample diversity, potentially impacting model generalizability across diverse clinical scenarios. Second, the framework demonstrates suboptimal performance in identifying “Trend” entities, which exhibit particularly complex linguistic patterns and high textual variability. Third, our relation extraction experiments reveal inefficiencies in processing complex relations like “STr”, highlighting fundamental constraints of purely rule-based approaches. Fourth, while the annotation guidelines developed in this study were rigorously refined through multiple iterations involving two licensed TCM physicians and two TCM master’s candidates, we recognize that our team’s expertise alone is insufficient to ensure the guidelines’ comprehensiveness and broad applicability. Fifth, the effectiveness of the fine-grained corpus developed in this study in terms of standardization has been verified; however, its effect in actual TCM clinical practice is only supported by theory [8, 9] and has not yet undergone experimental validation.

To address these challenges, we propose a multi-pronged research agenda: (1) expanding to multi-source medical data to enhance diversity and representativeness, (2) optimizing entity recognition through fine-grained segmentation and description simplification for challenging categories like “Trend” (3), developing hybrid relation extraction models that combine rule-based methods with deep learning architectures, particularly focusing on low-resource scenarios [49], (4) validating and refining the annotation guidelines by incorporating more TCM experts from diverse practice settings to ensure scholarly rigor and clinical relevance across varied clinical environments, and (5) we plan to convene TCM clinical experts to conduct clinical application verification experiments on the extracted data in accordance with current TCM diagnostic standards, thereby systematically evaluating its diagnostic value and application potential in real-world diagnosis and treatment scenarios.

## Conclusion

Corpus construction is a fundamental and essential task for discovering valuable TCM knowledge. This study proposes a method suitable for constructing a standardized output of symptom information in TCM EMRs. The final NER and RE experiments and the standardized output results indicate that the method proposed in this study is effective. The constructed corpus has a high degree of standardization, which not only provides efficient and reliable data support for the standardized processing and accurate analysis of symptom information but also lays a solid foundation for the construction of high-quality corpora and subsequent research, thus enabling the efficient extraction and application of TCM symptom information.

In the future, with the rapid development of large language models and self-supervised learning technologies, integrating these advanced technologies will enable more automated and efficient standardized output, further optimizing the extraction and standardization process of TCM symptom information. This will lay a solid foundation for the modernization of TCM applications and promote its widespread use in intelligent diagnostic systems, health management, personalized medicine, and other fields.

## Electronic supplementary material

Below is the link to the electronic supplementary material.

Supplementary Material 1

## Data Availability

As the raw analytical data were derived from real medical records, which involve patient privacy and ethics, this part of the data could not be made available to the public. However, the normalized database and all analytical code developed for this study are available from the corresponding author upon reasonable request for research purposes.
