# Supplementary material for: A framework for normalized extraction of fine-grained traditional Chinese medicine symptom entities and relations
Source: BMC Med Inform Decis Mak. 2025 Dec 6;25:441. doi: 10.1186/s12911-025-03257-4 (PMC12683873; doi:10.1186/s12911-025-03257-4)
Supplement: Supplementary file 1 — Supplementary Material 1 [file 12911_2025_3257_MOESM1_ESM.docx]

# Guidelines for Fine-Grained Annotation of Chinese Medicine Symptoms

## 1 General rules

### 1.1 Entity annotation principles

According to the needs of TCM clinical, combined with natural language processing technology, the fine-grained extraction of TCM clinical information is implemented. Due to the development and summary of traditional Chinese medicine, the description of Chinese medicine symptom information is composite, summary and concise, which makes the modern naming entity technology cause certain troubles to the extraction of Chinese medicine symptoms and dialectics, and there is even a black-box problem under the dialectics based on the large language model. In order to meet the needs of standardised symptom extraction and accurate diagnosis and treatment, a symptom description structure of "position + state" is proposed, in which the description of a piece of clinical history information is split into three dimensions, temporal, symptomatic, and severity, which are subdivided into 12 types of entities and 10 types of relations; this is used to accurately capture all aspects of the symptom information.

In addition to following the fine-grained annotation guidelines developed, this study also follows the following basic principles for entity annotation:

1. No overlapping annotation; an entity cannot be annotated as two or more entity types;
2. No nested annotation; the appropriate entity type is selected according to the contextual semantics when annotating and an entity cannot be annotated again in an entity;
3. Maximize coverage annotation; entities with progressive relations within the same entity type implement maximize coverage annotation. For example, in "左手小指疼痛 (pain in the little finger of the left hand)", "手小指 (little finger of the hand)" is the main position with a progressive relation, so the whole is annotated as the "Position_Primary".

### 1.2 Relation annotation principles

Relations are relatively high-level semantics, so ideally, annotating symptom relations relies on both the correctness of the text and its consistency with external knowledge. However, real clinical texts contain a large number of non-negligible typos, grammatical errors, and content errors that make symptom relations difficult to interpret or lead to conflicts between textual meaning and external knowledge. As mentioned earlier, annotation should follow text literalness more preferentially than external knowledge margins. Thus, as long as the text clearly presents symptom-information relations, the annotator should annotate them accordingly; otherwise, the annotator may try to infer the implied relations from the text with the help of his medical knowledge. The following principles of relation annotation are followed:

1. "State" as subject: except for the relation between "position" and "state", the rest of the relation takes "state" as subject;
2. Progressive annotation: In the "position" relation annotation, follow the progressive order of annotation from "Position_Subordinate" → "Position_Primary" → "Position_Scope".

### 1.3 Fine-grained entity and relation definitions

For the complex and diverse clinical information extraction in TCM, in order to ensure the coverage and accuracy of the annotation as well as to lay a firm foundation for the identification and treatment of TCM, the following entity labels are set through the research and analysis, as shown in Table 1.

Table 1 The definitions of the 12 types of entities in this study and their examples (Abbr: Abbreviation. In the examples, the entities are in bold font.)

| **Entity type** | **Abbr** | **Definition** | **Examples** |
| --- | --- | --- | --- |
| Symptoms_Single | Symp_S | A symptom element that can still independently express the meaning of a symptom when the position is omitted; includes highly condensed symptom descriptions or paraphilic expressions in TCM terminology. | **咳嗽**,发热,恶心(cough, fever, nausea) |
| Position_Scope | Pos_SCP | Describe and limit the scope of the primary position. | **左侧**头痛(left-sided headache) |
| Position_Primary | Pos_Pri | The major positional components of a symptom, including body parts, physiological products in physical position, and mental, spiritual, and respiratory in conceptual position. | **头**痛,神志不清,舌红,脉细(headache, mental confusion, red tongue, thready pulse) |
| Position_Subordinate | Pos_Sub | Between the primary position and state, it describes various aspects of the primary position or state, including quantity, color, texture, visual objects, joints, skin, and so on. | 尿量多(excessive urination) |
| State | State | A symptom's manifestation within a specific position, including but not limited to the elements of the symptom. | 大便干,头痛,睡眠差(dry stools, headaches, poor sleep) |
| Time | Time | Describes the time when the symptom set occurs. | **昨日早晨**呕吐(vomiting yesterday morning) |
| Condition | Cond | Describes the specific time or conditions triggered by a single or a few symptoms. | **活动时**气促(shortness of breath during activity) |
| Negation | Neg | Describes the absence of symptoms, used to explicitly indicate that certain symptoms have not occurred. | **无**恶心,未见头痛(no nausea, no headache) |
| Frequency | Freq | Describes the frequency or periodicity of the symptom occurrence. | 头痛反复,呕吐一天一次(recurrent headaches, vomiting once a day) |
| Severity_Qualitative | Sev_Qual | Expressing symptom severity through textual descriptions without involving specific numerical values or metrics. | 咳嗽严重，轻微鼻塞(severe cough, slight nasal congestion) |
| Severity_Quantitative | Sev_Quant | Describe symptom severity through specific numerical values or metrics. | 发热，39℃(Fever, 39℃) |
| Trend | Trend | Describe tendencies in symptoms, including whether the condition is getting progressively worse, better, or remaining stable. | 腹痛加重，气促减轻(increasing abdominal pain，decreasing shortness of breath) |

Table 2 The 10 types of relations and their examples (In the examples, the subject is in bold and the object is underlined.)

| **Relation type** | **Abbr** | **Examples** |
| --- | --- | --- |
| (State/Pos_Pri/Pos_Sub)_Pos_SCP | SSCP | 左侧**胸部**(left side of chest) |
| (State/Pos_Sub)_Pos_Pri | SPri | 神志**不清**( mental confusion) |
| (State)_Pos_Sub | SSub | 视物**模糊**(blurred vision) |
| (State/Symp_S)_Time | STi | 昨日**呕吐**(vomiting yesterday) |
| (State/Symp_S)_Cond | SC | 行走时**疼痛**(pain while walking) |
| (State/Symp_S)_Neg | SN | 无**恶心**(no nausea) |
| (State/Symp_S)_Freq | SF | **疼痛**反复(recurrent pain) |
| (State/Symp_S)_Sev_Qual | SQual | 轻微**咳嗽**(slight cough) |
| (State/Symp_S)_Sev_Quant | SQuant | **发热**，39℃(fever, 39℃) |
| (State/Symp_S)_Trend | STr | **咳嗽**加重(cough aggravation) |

### 1.4 Detailed Explanation of Entity Types

Symptoms_Single. This entity is divided into two categories: The first refers to certain symptoms that, in the process of language expression and evolution, omit the physical or conceptual position where they occur, and are described only by their state without causing ambiguity; the second refers to highly condensed symptom descriptions in Chinese medicine terminology, where some terms summarize multiple symptoms but cannot be split based solely on the words.

State: This refers to the manifestation of a symptom or sign within a specific position. It typically involves specific physiological, pathological, or disease characteristics and describes the state of a parameter during the disease or physiological process. This concept emphasizes the manifestation within a specific position. Includes, but is not limited to, the symptom elements in the TCM Symptom Terminology reference book [1]. For different types of "Position_Primary", the description of the state may not always be a specific word; if the "Position_Primary" is normal physiological activity, the frequency of occurrence is usually described as the state; if it involves pathological substances, the color, quantity, or texture changes of the pathological substances are used to describe the state. Therefore, the state includes various types of words, and the description may change based on the focus of the semantic context.

Position_Primary: This refers to the subject described when a particular symptom occurs. To better understand the definition of position in this context, it is divided into physical position and conceptual position. This classification helps differentiate visible, concrete physical entities from abstract functional or mental entities. Physical position refers to parts of the body or physiological products that can be directly seen or touched. Conceptual position refers to intangible and abstract existences, such as functions, sensations, or mental activities.

Position_Subordinate: This refers to position descriptions that are attached to the "Position_Primary" and state. Lack of secondary position may sometimes lead to ambiguous clinical descriptions. The specific situation depends on the state, as it describes different aspects of the "Position_Primary" or state, including quantity, color, texture, vision, joints, skin, etc.

Position_Scope: This refers to the spatial description that limits the "Position_Primary" of the occurrence state. The relative spatial relationship is defined by terms like "left side, right side, bilateral, up, down, large, small," and so on, to delineate an area. It can be used to describe specific ranges or boundaries within the "Position_Primary", typically centered around the "Position_Primary" and using different range descriptors to form a limited region.

Time: This refers to the time point or time period when the symptoms first appear. Any clinically meaningful information following the time expression, whether it is Western medicine content or TCM content, should be annotated. This generally refers to any time-related word used in a medical event to describe the symptoms. It mainly includes dates, seasons, time periods, solar terms, and physiological cycles.

Condition: This refers to specific times or conditions that trigger one or more symptoms. These conditions may be inherent changes in physiological, metabolic, or immune systems, or external factors such as the environment, emotions, or behavioral stimuli.

Negation: This refers to terms used to deny the existence of symptoms in medical event descriptions, clearly indicating that certain symptoms, signs, or diseases have not occurred. It is commonly used in clinical practice to exclude specific diagnoses or clinical manifestations.

Frequency: This refers to the frequency or periodicity of the occurrence of a symptom, disease, or event. It describes the frequency or repetition of a symptom or sign occurring within a certain period.

Severity_Qualitative: This refers to the clinical manifestation of the severity of a symptom or disease, evaluated through descriptive language or classification, without involving specific numerical values or calculations.

Severity_Quantitative: This refers to describing the clinical manifestation of the severity of a symptom or disease by measuring its severity with specific numerical values, indicators, or mathematical formulas.

Trend: This refers to the trend of change in a symptom or disease, including whether the condition is gradually worsening, improving, or remaining stable. Based on clinical descriptions, the development trend is divided into two types: one that directly describes whether a symptom is worsening or improving, and another that describes a condition with a possibility of worsening.

## 2 Detailed example of annotation

### 2.1 "Symptoms_Single"

"Symptoms_Single" includ symptoms whose position was omitted during the evolution of language without affecting comprehension and highly condensed terms in TCM, etc. Examples include "咳嗽(coughing)", "呕吐(vomiting)", "恶心(nausea)", "不喜言语(not willing to speak)", and "天旋地转感((sensation of the world spinning)".

Annotation example:

①**咳嗽[Symp_S],恶心[Symp_S],呕吐[Symp_S]**

②**不喜言语[Symp_S],天旋地转感[Symp_S]**

### 2.2 "State"

"State" indicates the manifestation of a symptom or sign in a given position. It usually involves a specific feature of physiology, pathology or disease, and is a stateful description of a parameter in the course of a disease or physiological activity. Examples include "痛(pain)", "闷(stuffiness)", "肿胀(swelling)", and "脱出(detachment)".

Annotation example:

①晨起时头**痛[State]**(headache in the morning)，胸**闷[State]**(chest stuffiness)，

②上厕所时肿物**脱出[State]**(a mass detached when going to the toilet)

### 2.3 "Position_Primary"

The "Position_Primary" is the subject of the position described when a symptom occurs. It is divided into physical position, which refers to the physical parts that can be directly seen and touched, such as parts of the human body and physiological products, and conceptual position, which refers to the intangible and abstract existence, usually including functions, states, sensations or mental activities.

Annotation example:

①双下**肢[Pos_Pri]**麻木(numbness of both lower limbs)，**舌[Pos_Pri]**红(red tongue)，**苔[Pos_Pri]**黄腻(yellowish and greasy tongue coating)，**脉[Pos_Pri]**弦滑(stringy and smooth pulse)。

②**神[Pos_Pri]**清(spirit is clear)，**精神[Pos_Pri]**可(mentally fine)，**行走[Pos_Pri]**不利(walking is unfavourable)，

### 2.4 "Position_Subordinate"

"Position_Subordinate" are positional descriptions that exist in affiliation with "Position_Primary" and "state", and the absence of "Position_Subordinate" can sometimes result in ambiguity in the presentation of clinical information, depending on the state.

Annotation example:

①右耳**听力[Pos_Sub]**下降(right ear hearing loss)，双眼**视物[Pos_Sub]**模糊(blurred vision in both eyes)

②尿**量[Pos_Sub]**减少(decrease in urine output)，尿**次数[Pos_Sub]**增多(increase in the number of times of urinating)

### 2.2 "Position_Scope"

"Position_Scope" is a description of the extent of the limitations of the "Position_Primary", such as "left, right, bilateral, up, down, big, small", etc.

Annotation example:

①**双[Pos_SCP]**手肘肩关节疼痛(Pain in elbow and shoulder joints of both hands, improving with movement)，**双[Pos_SCP]下[Pos_SCP]**肢小腿感觉麻木(numbness in lower legs of both limbs)

②**大[Pos_SCP]**便干(dry stools)，**小[Pos_SCP]**便黄(yellow urine)

### 2.6 "Time"

"Time" is the point or period of time when a symptom, disease or event begins to appear, which mainly includes the date, season, hour, festival, etc.

Annotation example:

①**2023年1月3日[Time]**(3 January 2023,)，**10年前[Time]**(10 years ago)

②**昨日早晨[Time]**(yesterday morning)，**今天下午[Time]**(this afternoon)

### 2.7 "Condition"

"Condition" is a trigger or prerequisite for the manifestation of a symptom or disease. These conditions may be internal physiological, metabolic, or immune system changes, or external environmental, emotional, or behavioural stimuli.Annotation example:

①**摔倒[Cond]**致右髋部疼痛(pain in the right hip from a fall)，下肢被**蚊子叮咬Cond**后出现一小皮疹(a small rash on the lower limb from a mosquito bite)

### 2.8 "Negation"

"Negation" is a term used in the description of a medical event to negate the presence of symptoms, and is used to explicitly state that certain signs, symptoms or diseases are not present.

Annotation example:

①**无[Neg]**恶心呕吐(no nausea or vomiting)，**未见[Neg]**咳嗽(no cough)

### 2.9 "Frequency"

"Frequency" is the frequency or periodicity with which a symptom, disease or event occurs. Describes how often a symptom or sign occurs or is repeated over a period of time.

Annotation example:

①**反复[Freq]**排尿不畅(repeated dysuria)

②双下肢**间歇性[Freq]**疼痛(intermittent pain in both lower limbs)

③**近2月以来[Freq]**体重下降约30斤(weight loss of about 30kg in the last 2 months)

### 2.10 "Severity_Qualitative"

"Severity_Qualitative" is the degree of clinical manifestation of a symptom or disease, assessing the severity of a symptom through descriptive language or categorisation to improve the accuracy of the description of the symptom without reference to a specific numerical value or calculation.

Annotation example:

①左下肢水肿**明显[Sev_Qual]**，**按之凹陷不起[Sev_Qual]**(left lower limb oedema is obvious, press the depression can not afford)

②尿中有**大量[Sev_Qual]**泡沫，**久久不散[Sev_Qual]**(a large amount of foam in the urine, which does not dissipate for a long time.)

### 2.11 "Severity_Quantitative"

"Severity_Quantitativ" is a measure of the severity of a symptom by means of a specific numerical value, indicator or mathematical formula when describing the degree of clinical manifestation of a symptom or disease.

Annotation example:

①左上肢皮肤肿物**大小3mm*2mm*3mm[Sev_Quant]**(size of skin swelling on left upper limb 3mm*2mm*3mm)

②体重下降**20kg[Sev_Quant]**(weight loss of 20kg)

### 2.12 "Trend"

"Trend" describes the tendency of a symptom or disease to change, including whether the condition is getting worse, getting better or remaining stable.

Annotation example:

①昏倒，**休息后可缓解[Trend]**(fainting, relieved by rest)

②三天前头晕**加重[Trend]**(dizziness worsened three days ago)

## 3 Classification obfuscation processing

### 3.1 "State" versus "Severity" and "frequency"

In medical terminology, the description of symptoms is not a single dimension, for different symptoms, the subject of the description is different, and its corresponding state is also different types of words. In the case of "headache, once a day", "once a day" is the frequency, but in the case of "urinate four times a day", "four times a day" is the state of the physiological act of urination.

### 3.2 "Time" versus "Condition"

The major difference between "Time" and "Condition" is that "Time" is the time when a set of different symptoms appear, whereas condition is a special point in time or a trigger that leads to the appearance of a particular symptom. For example, "三个月前出现了咳嗽、发热、恶寒，夜尿多(three months ago, there was a cough, fever, chills, and urinating a lot at night)", in which "三个月前(three months ago)" is the time of occurrence, and "夜(night)" is the condition of the occurrence of polyuria.

## Refrence

[1] Wang, Z. G. (2015). Basis of Standardization of Pathologic terms People's Medical Publishing House.
